
JOURNAL INFORMATION
==============================
NLM Title Abbreviation: J Can Health Libr Assoc
Journal ID: J Can Health Libr Assoc
NLM Journal ID: 101229936
Journal ID: JCHLA

The Journal of the Canadian Health Libraries Association

EISSN: 1708-6892
Publisher: Canadian Health Libraries Association / Association des bibliotèques de la santé du Canada

ARTICLE INFORMATION
==============================
PMCID: PMC12352442
DOI: 10.29173/jchla29896
Article ID: JCHLA-46-066
Article version: 1
Subjects: Abstracts

CHLA 2025 CONFERENCE LIGHTNING TALKS / ABSC CONGRÈS 2025 PRÉSENTATIONS ÉCLAIR

Electronic publication date: 2025 Aug 1
Publication date: 2025 Aug
Volume: 46
Issue: 2
First page: 66
Last page: 74
Copyright: © Fournier
Copyright year: 2025
License: This article is distributed under a Creative Commons Attribution License: https://creativecommons.org/licenses/by/4.0/
License URL: https://creativecommons.org/licenses/by/4.0/

==============================

## LT1. Building a better future together by assessing our systematic review service

Laurel Scheinfeld, Sunny Chung, Jessica Koos, Michael Huang
Stony Brook University

**Introduction:** We previously presented a paper on new processes and documents implemented for our library's systematic review service at the annual CHLA conference in 2023. A task force of health sciences librarians had aimed to formalize our systematic review service to make it more standard, transparent, and to cut down on zombie (uncompleted) reviews. Now that we are two years into using the new procedures and forms, we have decided to assess the model. **Methods/Description/Discussion:** We will share feedback on use of the new intake form, MOU document, and protocol template that were developed and we'll discuss the edits to the forms and procedures we are considering based on this feedback. We will also review our positive and negative experiences using a project management software program to store and share data from our knowledge synthesis projects. Future plans for analyzing quantitative data and obtaining patron feedback will also be discussed.

## LT2. Chapter by chapter: book club conversations with the CEO

Jeanna Hough
Halton Healthcare

**Introduction:** The Clinical Librarian, CEO, and Leadership Institute partnered to create a bi-annual book club that fosters collaboration, leadership development, and organizational growth. Description: This innovative biannual book club brings together the hospital librarian, CEO, and a small group of staff, sponsored by the Leadership Institute to explore critical topics at the intersection of leadership, information literacy, and diversity, equity, and inclusion (DEI). Each session features a carefully selected book that challenges perspectives, inspires dialogue, and promotes personal and professional growth. Guided by the CEO's strategic vision and facilitated by the librarian's expertise, participants engage in thought-provoking discussions that enhance leadership skills, foster inclusivity, and empower informed decision-making. This collaborative initiative cultivates a learning community, aligning organizational goals to build stronger connections across our hospital teams, transforming reading into a catalyst for impactful change. **Outcomes:** The inaugural test year of the book club covered two books across winter and fall sessions, with a total of four meetings. Initially offering one session per season, participant demand was so high that slots filled within minutes, prompting the addition of more sessions. **Discussion:** Given its success, the book club is now permanent, with plans to expand across all three hospital sites while remaining in-person. Participant feedback has been extremely positive, highlighting the value of meaningful discussions, leadership growth, and the connections fostered through the program. This strong foundation sets the stage for continued impact and broader engagement. The program has enhanced the Library's visibility hospital-wide and is forging new connections for the Librarian across all levels of the organization.

## LT3. Cleaning up duplicate clinical trial records like a pro

**Background:** Searching trial registries is a mandatory item in the Cochrane MECIR guidelines, and two registries, the World Health Organization International Clinical Trials Registry Platform (ICTRP) and ClinicalTrials.Gov (CTG), are specifically recommended in the Cochrane Handbook of Systematic Reviews of Interventions (Chapter 4). As ICTRP contains records from CTG, a search of both resources will lead to duplicate records. Automated tools and existing published deduplication methods are not suited to deduplicating registry records as they are based on comparison of fields such as title, author, journal name, year, and other metadata fields that are common to bibliographic records. Some of these fields are not commonly used in registry records, and even fields such as the title can differ across the same trial's record from various sources. **Methods:** This lightning talk will demonstrate the problem, including why software like Covidence fails at detecting duplicates between ICTRP and CTG. We will also present a method for deduplication of registry records in EndNote (desktop) using the unique study ID field as the sole deduplication parameter. **Conclusion:** This method is particularly suited for librarians supporting reviews of interventions who have to search multiple resources that contain trials records (CTG, ICTRP, and even Cochrane CENTRAL).

## LT4. Compounding drugs data: introducing a drug terms tool for knowledge synthesis projects

Tyler Ostapyk
University of Manitoba

**Introduction:** For knowledge synthesis projects concerning pharmaceutical interventions, the development of a comprehensive search strategy generally requires the identification of various brand names and synonyms used for a particular drug. Building a list of these terms can take a substantial amount of time and effort, and often requires the consultation of numerous thesauri and authoritative sources. To save searchers time when building their list of terms, the author has developed a Python-based tool that queries various data sources (MeSH, RXNorm, Wikidata, and PubChem) and produces a search string that can be directly input into bibliographic databases. **Description:** Using an HTML form, the searcher enters a drug name and specifies which data sources they would like to query. The tool then queries the selected data sources for the drug. If there is a match it identifies and retrieves available synonyms. The retrieved terms are then combined into a single search string that can be used in bibliographic databases such as Ovid Medline or CINAHL. **Outcomes:** Leveraging existing data sources, the tool can quickly generate search strings for specific drugs. These strings return a more comprehensive set of search results than single text word searches and generating the strings requires minimal effort. **Discussion:** Further work is needed to determine the effectiveness of the search strings generated by the tool, especially in comparison to manually created strings. Restrictive API access policies and fees have prevented the inclusion of some authoritative sources, which may limit the tool's effectiveness.

## LT5. Data sharing practices amongst original research articles published in hybrid vs open access medical librarianship journals

Eden A. Kinzel
University of Toronto

**Introduction:** As the open access (OA) movement progresses, more attention is being shifted not just to the article's availability but also the availability of data used or produced by the article; especially considering the persistence of the reproducibility crisis. Another concern might be if there is a difference in data sharing practices between articles published in hybrid journals as compared to open access journals. **Methods:** 322 research articles published between 2021 through 2024 in six medical librarianship journals (three hybrid and three open access) were analyzed. Though all articles categorized in the original/featured/research article sections were initially included in the sample, only the 200 articles which conducted data analysis were analyzed fully (program descriptions and other narrative articles were excluded). **Results:** Data availability statements were present in 56% of articles (29% of hybrid articles and 74% of OA articles) and data collection was reproducible in 55% of articles (47% hybrid, 60% OA). 30% of articles shared raw data (13% hybrid, 41% OA). When data was shared, 51% of articles analyzed shared within the appendix (38% hybrid, 60% OA), 21% within OSF (15% hybrid, 25% OA), and 14% within an institutional repository (8% hybrid, 18% OA). **Discussion:** The presentation intends to discuss differences in data sharing practices between articles published in hybrid vs open access medical librarianship journals. Results of this lightning talk may inform researchers' information retrieval practices when searching for datasets to analyze or projects to reproduce.

## LT6. Development of a full-text search strategy for CMAJ's scoping review on content about First Nations, Inuit and Métis people and anti-Indigenous racism

**Introduction:** As part of its reconciliation journey, CMAJ seeks to understand the impact and extent of Indigenous-specific racist content published in the journal. To support a scoping review on anti-Indigenous racism, librarians developed an extensive search strategy to identify all articles about or related to First Nations, Inuit and Métis people in Canada in the published contents of CMAJ from January 1911 to August 2024. In addition to Ovid MEDLINE (MEDLINE), PubMed Central (PMC) was selected for its full archive of CMAJ. **Description:** Full-text search was necessary to retrieve contents not indexed in MEDLINE and earlier contents when journal and database indexing was limited. A comprehensive list of search terms was assembled using existing published search filters, and by consulting subject matter experts and terminology guides on Indigenous Peoples in Canada. Extensive testing searches led to our final full-text search strategy that includes approximately 1200 keywords and the development of an internal CMAJ search aid that documents lessons learned about search platforms, syntax and more. **Outcomes:** A total of 7540 and 774 references were found in PMC and MEDLINE, respectively. After deduplication of the combined results, 7580 unique references were retrieved. Most references retrieved in MEDLINE were found in PMC. **Discussions:** This is a unique search where a scoping review involves full-text search. At the time of writing, the CMAJ team is still screening search results. Given our sensitive search strategy, a large number of irrelevant references are anticipated. We'll assess the effectiveness of our search strategy once the screening is complete.

## LT7. Documenting the shift: how researchers report generative AI in search methodologies for evidence synthesis

**Objective:** This lightning talk will provide a brief description of a scoping review designed to identify the extent to which generative AI is being used in the search methods of evidence synthesis reviews. We will compare tools and strategies used by review authors, and extract details on the level of reporting. **Methods:** The JBI scoping review methodology will guide the conduct of this review. After calibration exercises on screening and data extraction were completed, an a priori protocol was published on OSF

Registries. To be eligible for inclusion, a review must be a known type of evidence synthesis, and authors must have either used a generative-AI powered tool to develop database search strategies or used an AI search engine to locate references directly. We will search from Jan 2022 to present: Ovid Medline, Ovid Embase, Ovid PsycINFO, EBSCO CINAHL, EBSCO ERIC, ProQuest Sociological Abstracts, Elsevier Scopus, and Clarivate Web of Science Core Collection. We will conduct a supplementary full-text search in EBSCO MEDLINE, EBSCO CINAHL and Lens.org. We will independently screen in two stages in Covidence; disagreements will be resolved by consensus and discussion. We will extract study characteristics; characteristics related to the method, type of chatbot or AI search engine used; description of search method; and search reporting elements. The results will be presented in tables, accompanied by descriptive summaries. **Results and Discussion:** This project will provide insights into the adoption and reporting of generative AI tools in KS searches.

## LT8. How student librarians can support Indigenous health knowledge translation: a program proposal

Caprice Pybus[1], Coco Chen[2], Rebecca Ardron[3], Natalie Reddy[2]
[1]University Canada West, [2]University of British Columbia, [3]Alexander College

**Abstract:** Within health librarianship, there exists an immediate need for both students and health sciences librarians to connect with the First Nations Health Authority (FNHA) through sustained outreach. Utilizing the FNHA's strategic plan as a starting point, we propose a long-term collaboration with UBC's School of Information (iSchool) to ensure that Indigenous communities maintain their authority over the implementation of traditional health practices. We present this framework for LIS students as a Professional Experience (PE) project, supervised by a health sciences librarian, to work alongside Indigenous Peoples towards the goal of creating a long-lasting partnership for traditional Indigenous health knowledge mobilization. **Description:** With the guidance of the FNHA, iSchool students would assist with the creation of information resources and tools such as LibGuides about traditional medicines, health resources, and practitioners for use within community contexts. Where possible, the program could assist in knowledge transfer from Elder to information guides, and when information is sensitive, we would assist in creating templates for information retrieval to maintain consistency. **Outcomes:** The goal for the collaborating information professionals is not to extract this knowledge, but instead to facilitate knowledge translation and mobilization by and for these communities. The highest priority in these efforts is that the Indigenous communities we work with maintain their authority over and improve access to their information. **Discussion:** This proposal differs from existing library programs by collaborating more directly with Indigenous communities, working with knowledge and materials that may not be academically published, and building a space for limited access to culturally sensitive materials.

## LT9. Integrating Indigenous Ways of Knowing with citation guidelines in Canadian health sciences libraries: an environmental scan

Margaret Banka
University of Manitoba

**Introduction:** Though academic institutions strive to embrace Indigenous Ways of Knowing into research praxis, a true integration of Western and Indigenous values remains challenging and elusive. One manifestation of this issue is the treatment of Indigenous Knowledge sources in American Medical Association (AMA) and American Psychological Association (APA) citation styles, which are the primary citation styles used in health sciences research. Recently, there have been radical efforts by academic librarians in Canada to propose new, more inclusive templates for citing Indigenous Knowledge sources. This study seeks to evaluate this emerging practice across academic institutions in Canada to measure current practices in health sciences libraries. **Methods:** Publicly available AMA, APA, and Indigenous citation guides will be identified among 297 universities and colleges in Canada. For those institutions that demonstrate one or more of these citation guides, data will be extracted to determine how many and which libraries are circumventing APA and AMA guidelines on Indigenous Knowledge source citation, and what elements they are recommending. A frequency distribution table will be created in Excel to summarize trends across institutions in Canada. **Results:** Findings will be presented at the time of the conference as this research is currently in progress. **Discussion:** The data will provide valuable insight into which libraries across Canada are actively decolonizing academic citation practices, how they are doing it, and what is being done among health sciences libraries. The data also has the potential to inform health science librarians' own approach to and recommendations for citing Indigenous Knowledge sources.

## LT10. Lack of involvement of medical librarians/information specialists in systematic reviews submitted to a high-ranking medical journal: insights from an editorial board member and reviewer

**Background:** A rigorous literature search is essential for systematic reviews (SRs) quality. However, many medical journals lack the resources to thoroughly peer-review search methods and strategies. The extent of medical librarians/information specialists' (MLIS) involvement in developing literature searches is often unclear. **Methods:** As a former Cochrane information specialist and a researcher with over 20 years at various universities, I have served as an editorial board member and technical reviewer for a high-ranking medical journal A for 13 years. In this role, I critically assess all submitted SRs, including their search methods and strategies, using the PRISMA-S checklist, Cochrane guidelines, and the PRESS peer review checklist. This abstract summarizes my analysis of 90 SRs I reviewed for Journal A in 2024, documented prospectively without a second reviewer to maintain confidentiality. **Results:** Of 90 SRs, only 6 (7%) included an MLIS as a co-author, and 10 (11%) mentioned MLIS assistance, with only three MLIS names formally acknowledged. Although most SRs claimed PRISMA compliance, 18 (20%) failed to submit complete search strategies for at least one database. I provided comments on search methods (1-9 points, median 5) for each SR that received an editorial decision). 20 SRs (22%) were accepted with major revisions. All of the 19 SRs that had submitted a revised version incorporated the suggested revisions; 4 (21%) of them included more studies following an updated search. **Conclusion:** MLIS involvement in SRs is limited and often inadequately acknowledged, underscoring the importance of rigorous peer review at all stages.

## LT11. Librarian roles in environmental sustainability: update of a strategic plan

Suzanne Fricke
Washington State University

**Program Introduction:** Health Science academic programs are increasingly seeking to integrate environmental sustainability into their curricula and core principles. This presentation explores how librarians can contribute effectively to this initiative. **Description:** A community-based medical school with a mission to serve rural health aimed to embed environmental sustainability values and practices into its strategic plan. This broad concept encompasses climate resilience, equity, and local practices and purchases. Faculty began reviewing approaches taken by other institutions, however the college found that it needed to align its plan with the broader health science campus and the larger multi-campus public university system. Recognizing that the project's goals required evidence synthesis, the medical library proactively offered support. **Outcomes:** The medical librarian collaborated with a faculty member to complete a search for organizational standards, existing plans at comparable institutions, and scholarly publications about environmental sustainability in medical school goal setting. The team utilized an artificial intelligence assistant in part to align with broader campus and institution plans. Key environmental performance indicators identified in the review will be presented internally to inform gap analysis and benchmarking. **Discussion:** Evidence synthesis projects that inform internal decision-making processes enable the library to demonstrate its value to the community and highlight the range of services it can offer to health science faculty.

### LT12. Pirates vs paywalls: preliminary investigation into the utility of Sci-Hub download logs for identifying trends in user behaviour

Emma S. Garlock
University of Ottawa

**Introduction:** Sci-Hub is a well-known pirate repository that allows users to circumvent paywalls and download academic articles relating to various health sciences and STEM subjects. While Sci-Hub may increase access to information in a timely manner and may flag issues in established publishing practices, there are ethical, legal and security risks associated with the platform's use. This research uses data made public by Sci-Hub to better understand Canadian Sci-Hub user behaviour, which can help inform potential approaches for discussing Sci-Hub usage with users in need of health sciences and biomedical information. **Methods:** This research analyzed the Sci-Hub download log for 2017. IP information was used to identify Canadian downloads. Other information analyzed includes the date of download, user city, and DOI of the accessed article. The DOIs of top articles were loaded into Zotero to retrieve publication dates and titles for further analysis. **Results:** Results will showcase top Canadian cities for Sci-Hub use, temporal trends for 2017, and bibliographic information of frequently accessed articles. **Discussion:** This research provides some of the first data-driven insights into Sci-Hub user behaviour in a Canadian context. Currently, only the 2017 data is publicly available, but Sci-Hub is clear about its intentions to make full download logs available in the future. The preliminary analyses shown here provide a blueprint for others who may be interested in conducting their own analyses on Sci-Hub usage in their own contexts.

### LT13. Repair cafe: a space for community and climate action at UBC's Woodward Library

While health libraries are known as places of learning for faculty and students in science and medicine, they can also be spaces for bonding and skill sharing for all community members who use the library. UBC's Woodward Library embodied this mentality in November 2024 and February 2025, when we hosted our Repair Cafes. Hosted by UBC Library's Library Climate Action Team as programs associated with British Columbia Library Association's Climate Action Week and UBC's Climate Emergency Week events, the repair cafes were spaces in which students, staff, faculty, and community members were welcomed to bring broken electronics and clothing items and learn to repair them. The intention behind this initiative was not only to provide a repair service, but to encourage participants to learn to do their own repairs, empowering them to make more sustainable choices in the future. Drawing on local talent, volunteers were on hand to assist participants with hand and machine sewing, darning, and repair of small electronics. While attendees waited for their turn at the repair table, they were able to make reusable beeswax food wraps, attempt a climate-themed Lego challenge, check out a mending book display, or have a free treat. This lighting talk will discuss the benefits we saw from this event and the challenges we encountered. It will conclude with a brief discussion of how community engagement events related to collaborative learning and climate change fit in with the ethos of health and science academic libraries.

### LT14. Reparative description and classification in medical libraries

**Background:** Medical libraries can play a significant role supporting reconciliation through reparative description and classification practices. This lightning talk will showcase our group's collaborative efforts to address systemic bias in cataloguing systems by engaging in decolonization initiatives in academic and health authority library settings. Our project highlights our work to implement recommendation #5 of the CFLA-FCAB Truth and Reconciliation Report – the call to "[d]ecolonize access and classification by addressing the structural biases in existing schemes of knowledge organization and information retrieval" [1]. **Project Description:** Our project involves two key areas: revising harmful and outdated subject headings and adapting classification systems. The first component, reparative description, involves replacing harmful Library of Congress Subject Heading (LCSH) and Medical Subject Headings (MeSH) by integrating interim vocabularies such as those by the Manitoba Archival Information Network (MAIN) and the Greater Victoria Public Library (GVPL). The second component centers on adapting Métis-settler Librarian, Ashley Edwards's, modification of Kahnawá:ke Librarian, Brian Deer's Classification (BDC) system in a medical library setting. **Outcomes:** Reparative description and classification is an ongoing and iterative process. While key milestones include the integration of inclusive vocabularies and our initial adaptation of BDC, the work is far from complete. Future goals involve expanding community engagement to guide collection development practices, with the recognition these efforts must remain flexible and responsive to ongoing changes in the decolonization of description and classification. **Conclusion:** This lightning talk will encourage attendees to collaborate to advance reparative cataloguing practices, fostering a commitment to decolonizing libraries and building inclusive futures together.

## References

1. Callison C, chair; Canadian Federation of Library Associations. Truth and reconciliation report and recommendations [Internet]. Canadian Federation of Library Associations; 2018 [cited 2025 Jan 17]. 87 p. Available from: http://cfla-fcab.ca/wp-content/uploads/2018/10/Truth-and-Reconciliation-Committee-Report-and-Recommendations-ISBN1.pdf.

### LT15. Supporting physician assistant students: a search template for success

Margaret Banka, Caroline Monnin, Carol Cooke
University of Manitoba

**Introduction:** Physician assistant (PA) students present unique challenges and opportunities for support in a health sciences academic library. This cohort often comprises of learners with diverse educational backgrounds, ranging from undergraduate to advanced degrees. In Fall 2024, the University of Manitoba physician assistant program significantly expanded, doubling its enrollment. As part of their academic journey, every student must complete a capstone project, which requires meeting with a librarian to support an in-depth literature review. To meet this growing demand and enhance the effectiveness of librarian consultations, a search template form was developed. **Description:** The search template was designed as a step-by-step worksheet to provide help in developing a search in a health sciences database. The template discusses the important components of the search strategy, including concepts, keywords, controlled vocabulary, database syntax, and links to external resources at every step. **Outcomes:** This tool was distributed to PA students prior to the librarian consultation to provide a structured guide for building complex searches in health sciences databases. The aim was not only to equip students with essential skills for conducting systematic searches but also to facilitate more productive consultations with librarians by creating a shared framework for discussions. With the increase in students and diminished librarian capacity, the template became instrumental to adequately supporting this program. **Discussion:** In this presentation, we will share how the search template form has been integrated into our instructional and consultation practices, providing tailored support to physician assistant students as they navigate the complexities of their capstone projects.

### LT16. The check tag cliff: a rapid evaluation of check tags over time in Medline

Nicole Askin
University of Manitoba

**Introduction:** Check tags in PubMed/Medline are used to quickly identify key features of studies and study subjects, such as age groups. They are frequently incorporated in search strategies. However, such searches are reliant on check tags being dependable in their application. This study sought to evaluate some irregularities the author noticed around check tags. **Method:** Searches were run in Ovid Medline for a series of commonly used check tags in records of publications from 2015 to 2024. The absolute number of records employing these tags and the proportion of records employing these tags was then compared to the results of similar searches in Embase, PsycINFO, and CINAHL. **Results:** There was a sharp drop in both absolute and proportional numbers of most check tags on Medline records published over this time period. For example, use of female/ decreased from 30% of Medline records for papers published in 2019 to only 10% of records for papers published in 2023. However, PsycINFO and Embase indexing patterns differed markedly. **Discussion:** the trends noted have significant implications for use of check tags for searching in PubMed or Medline. Results give insight into how indexing varies between PubMed/Medline and other commonly used biomedical databases.

### LT17. Towards a more connected health research ecosystem: a framework for interoperable health data commons

Alyssa Foote
World Data System

The Research Data Alliance (RDA) is a global network of experts that fosters collaboration on key data-related challenges, such as interoperability. The Global Open Research Commons (GORC) model is one example that provides a high-level framework for interoperability across diverse research commons. This presentation will introduce the Health Data Commons GORC Profile Working Group, which aims to adapt the GORC model for health data commons (HDCs). By developing a standardized metadata schema, this work will enhance the findability, accessibility, interoperability, and reusability (FAIR principles) of health data, ultimately supporting researchers, clinicians, and improving patient care. This work has the potential to significantly impact the health research ecosystem, including the vital role health librarians play in supporting data-driven research. This presentation will provide an overview of the working group's objectives and progress, and discuss the potential implications of this work for the future of health data sharing and discovery.

### LT18. Using Scopus for collections analysis in medicine

Kristen Romme

Memorial University of Newfoundland

**Introduction:** The aim of this study is to assess the utility of Scopus for learning more about the citing and publishing habits of faculty members in Medicine. **Methods:** I used Scopus to search for publications by Faculty of Medicine authors at my institution that were published between 2022-2024. I exported the bibliographic details for those publications, as well as the cited references. I am analyzing the data to learn the journals most frequently published in and cited, and the percentage of open access publications. I will then compare those findings with our existing collections to assess how well we are meeting faculty needs when it comes to research literature. **Results:** Preliminary results demonstrate that Scopus provides quick and relatively easy access to high-level insights about the sources published in and cited by faculty in Medicine. Coverage of journals and book chapters is excellent, but some Canadian journals and locally published documents are not indexed, along with some other sources. **Discussion:** Results from this study demonstrate that Scopus is a useful tool for simple collections analysis, and that it provides relatively thorough (though incomplete) coverage of the research output of our medical faculty. Future findings will reveal the relevance and usefulness of the Health Sciences Library collection for one of our key user groups. Findings may have implications for future collections decisions.

## LT19. Who's hot, who's not: changes to literature search requestors over time

Shannon Long, Vinny Gibson, Yvette Ipsaralexi
Vancouver Coastal Health

**Objective:** Health authority and hospital library services have long supported clinicians with evidence-informed decision-making and patient care decisions. In healthcare organizations this most often comes in the form of literature searching. In the past, hospital libraries often focused on physicians at a local acute site or small regional area, while different staff may have relied on library services provided by their professional associations. With the evolution of full-service regional library teams serving patrons across the entire continuum of care, increased movement toward online services and point of care tools, coupled with changes in healthcare staff makeup, has the primary clientele for library's literature search service changed? **Methods:** For ten years, library staff within our large regional health authority have systematically recorded information about patrons requesting literature searches. This includes job title, department, and category (frontline clinicians, physicians/surgeons/psychiatrists, educators/leaders, management/operations and corporate level). By collating and summarizing entries of user categories each year, we seek to identify annual trends and determine if there have been any significant changes in patron categories over a ten-year period. **Results:** In progress. **Conclusion:** By analyzing this data, we aim to answer some of the following questions: have literature search requestors' job categories changed over ten years? Is there a clear trend? Is it worthwhile to collect this data, and does it provide valuable information, given the time and processes required? By reviewing these findings we hope to use the results to inform the library's service planning, marketing, outreach, training, and collections.

JOURNAL INFORMATION
==============================
NLM Title Abbreviation: J Can Health Libr Assoc
Journal ID: J Can Health Libr Assoc
NLM Journal ID: 101229936
Journal ID: JCHLA

The Journal of the Canadian Health Libraries Association

EISSN: 1708-6892
Publisher: Canadian Health Libraries Association / Association des bibliotèques de la santé du Canada

ARTICLE INFORMATION
==============================
Subjects: LT = Lightning Talk

LT1. Building a better future together by assessing our systematic review service

Scheinfeld Laurel
Chung Sunny
Koos Jessica
Huang Michael
Stony Brook University

JOURNAL INFORMATION
==============================
NLM Title Abbreviation: J Can Health Libr Assoc
Journal ID: J Can Health Libr Assoc
NLM Journal ID: 101229936
Journal ID: JCHLA

The Journal of the Canadian Health Libraries Association

EISSN: 1708-6892
Publisher: Canadian Health Libraries Association / Association des bibliotèques de la santé du Canada

ARTICLE INFORMATION
==============================
Subjects: LT = Lightning Talk

LT2. Chapter by chapter: book club conversations with the CEO

Hough Jeanna
Halton Healthcare

JOURNAL INFORMATION
==============================
NLM Title Abbreviation: J Can Health Libr Assoc
Journal ID: J Can Health Libr Assoc
NLM Journal ID: 101229936
Journal ID: JCHLA

The Journal of the Canadian Health Libraries Association

EISSN: 1708-6892
Publisher: Canadian Health Libraries Association / Association des bibliotèques de la santé du Canada

ARTICLE INFORMATION
==============================
Subjects: LT = Lightning Talk

LT3. Cleaning up duplicate clinical trial records like a pro

Premji Zahra 1
Cooper Chris 2
1 University of Victoria,
2 University of Bristol

JOURNAL INFORMATION
==============================
NLM Title Abbreviation: J Can Health Libr Assoc
Journal ID: J Can Health Libr Assoc
NLM Journal ID: 101229936
Journal ID: JCHLA

The Journal of the Canadian Health Libraries Association

EISSN: 1708-6892
Publisher: Canadian Health Libraries Association / Association des bibliotèques de la santé du Canada

ARTICLE INFORMATION
==============================
Subjects: LT = Lightning Talk

LT4. Compounding drugs data: introducing a drug terms tool for knowledge synthesis projects

Ostapyk Tyler
University of Manitoba

JOURNAL INFORMATION
==============================
NLM Title Abbreviation: J Can Health Libr Assoc
Journal ID: J Can Health Libr Assoc
NLM Journal ID: 101229936
Journal ID: JCHLA

The Journal of the Canadian Health Libraries Association

EISSN: 1708-6892
Publisher: Canadian Health Libraries Association / Association des bibliotèques de la santé du Canada

ARTICLE INFORMATION
==============================
Subjects: LT = Lightning Talk

LT5. Data sharing practices amongst original research articles published in hybrid vs open access medical librarianship journals

Kinzel Eden A.
University of Toronto

JOURNAL INFORMATION
==============================
NLM Title Abbreviation: J Can Health Libr Assoc
Journal ID: J Can Health Libr Assoc
NLM Journal ID: 101229936
Journal ID: JCHLA

The Journal of the Canadian Health Libraries Association

EISSN: 1708-6892
Publisher: Canadian Health Libraries Association / Association des bibliotèques de la santé du Canada

ARTICLE INFORMATION
==============================
Subjects: LT = Lightning Talk

LT6. Development of a full-text search strategy for CMAJ's scoping review on content about First Nations, Inuit and Métis people and anti-Indigenous racism

Bai Nan
de Gannes-Marshall Renee
Canadian Medical Association

JOURNAL INFORMATION
==============================
NLM Title Abbreviation: J Can Health Libr Assoc
Journal ID: J Can Health Libr Assoc
NLM Journal ID: 101229936
Journal ID: JCHLA

The Journal of the Canadian Health Libraries Association

EISSN: 1708-6892
Publisher: Canadian Health Libraries Association / Association des bibliotèques de la santé du Canada

ARTICLE INFORMATION
==============================
Subjects: LT = Lightning Talk

LT7. Documenting the shift: how researchers report generative AI in search methodologies for evidence synthesis

Premji Zahra 1
Nekolaichuk Erica 2
Fuller Kaitlin 3
1 University of Victoria,
2 University of Toronto,
3 St. Francis Xavier University

JOURNAL INFORMATION
==============================
NLM Title Abbreviation: J Can Health Libr Assoc
Journal ID: J Can Health Libr Assoc
NLM Journal ID: 101229936
Journal ID: JCHLA

The Journal of the Canadian Health Libraries Association

EISSN: 1708-6892
Publisher: Canadian Health Libraries Association / Association des bibliotèques de la santé du Canada

ARTICLE INFORMATION
==============================
Subjects: LT = Lightning Talk

LT8. How student librarians can support Indigenous health knowledge translation: a program proposal

Pybus Caprice 1
Chen Coco 2
Ardron Rebecca 3
Reddy Natalie 2
1 University Canada West,
2 University of British Columbia,
3 Alexander College

JOURNAL INFORMATION
==============================
NLM Title Abbreviation: J Can Health Libr Assoc
Journal ID: J Can Health Libr Assoc
NLM Journal ID: 101229936
Journal ID: JCHLA

The Journal of the Canadian Health Libraries Association

EISSN: 1708-6892
Publisher: Canadian Health Libraries Association / Association des bibliotèques de la santé du Canada

ARTICLE INFORMATION
==============================
Subjects: LT = Lightning Talk

LT9. Integrating Indigenous Ways of Knowing with citation guidelines in Canadian health sciences libraries: an environmental scan

Banka Margaret
University of Manitoba

JOURNAL INFORMATION
==============================
NLM Title Abbreviation: J Can Health Libr Assoc
Journal ID: J Can Health Libr Assoc
NLM Journal ID: 101229936
Journal ID: JCHLA

The Journal of the Canadian Health Libraries Association

EISSN: 1708-6892
Publisher: Canadian Health Libraries Association / Association des bibliotèques de la santé du Canada

ARTICLE INFORMATION
==============================
Subjects: LT = Lightning Talk

LT10. Lack of involvement of medical librarians/information specialists in systematic reviews submitted to a high-ranking medical journal: insights from an editorial board member and reviewer

Yuan Yuhong 1 2 3
1 Western University,
2 London Health Sciences Centre,
3 McMaster University

JOURNAL INFORMATION
==============================
NLM Title Abbreviation: J Can Health Libr Assoc
Journal ID: J Can Health Libr Assoc
NLM Journal ID: 101229936
Journal ID: JCHLA

The Journal of the Canadian Health Libraries Association

EISSN: 1708-6892
Publisher: Canadian Health Libraries Association / Association des bibliotèques de la santé du Canada

ARTICLE INFORMATION
==============================
Subjects: LT = Lightning Talk

LT11. Librarian roles in environmental sustainability: update of a strategic plan

Fricke Suzanne
Washington State University

JOURNAL INFORMATION
==============================
NLM Title Abbreviation: J Can Health Libr Assoc
Journal ID: J Can Health Libr Assoc
NLM Journal ID: 101229936
Journal ID: JCHLA

The Journal of the Canadian Health Libraries Association

EISSN: 1708-6892
Publisher: Canadian Health Libraries Association / Association des bibliotèques de la santé du Canada

ARTICLE INFORMATION
==============================
Subjects: LT = Lightning Talk

LT12. Pirates vs paywalls: preliminary investigation into the utility of Sci-Hub download logs for identifying trends in user behaviour

Garlock Emma S.
University of Ottawa

JOURNAL INFORMATION
==============================
NLM Title Abbreviation: J Can Health Libr Assoc
Journal ID: J Can Health Libr Assoc
NLM Journal ID: 101229936
Journal ID: JCHLA

The Journal of the Canadian Health Libraries Association

EISSN: 1708-6892
Publisher: Canadian Health Libraries Association / Association des bibliotèques de la santé du Canada

ARTICLE INFORMATION
==============================
Subjects: LT = Lightning Talk

LT13. Repair cafe: a space for community and climate action at UBC's Woodward Library

Bradshaw Rachael
University of British Columbia

JOURNAL INFORMATION
==============================
NLM Title Abbreviation: J Can Health Libr Assoc
Journal ID: J Can Health Libr Assoc
NLM Journal ID: 101229936
Journal ID: JCHLA

The Journal of the Canadian Health Libraries Association

EISSN: 1708-6892
Publisher: Canadian Health Libraries Association / Association des bibliotèques de la santé du Canada

ARTICLE INFORMATION
==============================
Subjects: LT = Lightning Talk

LT14. Reparative description and classification in medical libraries

Caines Melissa 1
Brady-Randle Erin 2
1 Canadian College of Naturopathic Medicine,
2 Fraser Health

JOURNAL INFORMATION
==============================
NLM Title Abbreviation: J Can Health Libr Assoc
Journal ID: J Can Health Libr Assoc
NLM Journal ID: 101229936
Journal ID: JCHLA

The Journal of the Canadian Health Libraries Association

EISSN: 1708-6892
Publisher: Canadian Health Libraries Association / Association des bibliotèques de la santé du Canada

ARTICLE INFORMATION
==============================
Subjects: LT = Lightning Talk

LT15. Supporting physician assistant students: a search template for success

Banka Margaret
Monnin Caroline
Cooke Carol
University of Manitoba

JOURNAL INFORMATION
==============================
NLM Title Abbreviation: J Can Health Libr Assoc
Journal ID: J Can Health Libr Assoc
NLM Journal ID: 101229936
Journal ID: JCHLA

The Journal of the Canadian Health Libraries Association

EISSN: 1708-6892
Publisher: Canadian Health Libraries Association / Association des bibliotèques de la santé du Canada

ARTICLE INFORMATION
==============================
Subjects: LT = Lightning Talk

LT16. The check tag cliff: a rapid evaluation of check tags over time in Medline

Askin Nicole
University of Manitoba

JOURNAL INFORMATION
==============================
NLM Title Abbreviation: J Can Health Libr Assoc
Journal ID: J Can Health Libr Assoc
NLM Journal ID: 101229936
Journal ID: JCHLA

The Journal of the Canadian Health Libraries Association

EISSN: 1708-6892
Publisher: Canadian Health Libraries Association / Association des bibliotèques de la santé du Canada

ARTICLE INFORMATION
==============================
Subjects: LT = Lightning Talk

LT17. Towards a more connected health research ecosystem: a framework for interoperable health data commons

Foote Alyssa
World Data System

JOURNAL INFORMATION
==============================
NLM Title Abbreviation: J Can Health Libr Assoc
Journal ID: J Can Health Libr Assoc
NLM Journal ID: 101229936
Journal ID: JCHLA

The Journal of the Canadian Health Libraries Association

EISSN: 1708-6892
Publisher: Canadian Health Libraries Association / Association des bibliotèques de la santé du Canada

ARTICLE INFORMATION
==============================
Subjects: LT = Lightning Talk

LT18. Using Scopus for collections analysis in medicine

Romme Kristen
Memorial University of Newfoundland

JOURNAL INFORMATION
==============================
NLM Title Abbreviation: J Can Health Libr Assoc
Journal ID: J Can Health Libr Assoc
NLM Journal ID: 101229936
Journal ID: JCHLA

The Journal of the Canadian Health Libraries Association

EISSN: 1708-6892
Publisher: Canadian Health Libraries Association / Association des bibliotèques de la santé du Canada

ARTICLE INFORMATION
==============================
Subjects: LT = Lightning Talk

LT19. Who's hot, who's not: changes to literature search requestors over time

Long Shannon
Gibson Vinny
Ipsaralexi Yvette
Vancouver Coastal Health

